# Bioequivalence trials for the approval of generic drugs in Saudi Arabia: a descriptive analysis of design aspects

**DOI:** 10.1186/s12874-024-02207-4

**Published:** 2024-04-05

**Authors:** Turki A. Althunian, Bader R. Alzenaidy, Raseel A. Alroba, Ohoud A. Almadani, Fahad A. Alqahtani, Albatool A. Binajlan, Amal I. Almousa, Deema K. Alamr, Malak S. Al-Mofada, Nora Y. Alsaqer, Hessa A. Alarfaj, Abdulmohsen A. Bahlewa, Mohammed A. Alharbi, Ali M. Alhomaidan, Abdulaziz A. Alsuwyeh, Abdulmohsen A. Alsaleh

**Affiliations:** 1 Research Informatics Department, Saudi Food and Drug Authority, Riyadh, Saudi Arabia; 2 College of Medicine, Alfaisal University, Riyadh, Saudi Arabia; 3 Department of Pharmacokinetics, Saudi Food and Drug Authority, Riyadh, Saudi Arabia; 4 Executive Department of Research and Studies, Saudi Food and Drug Authority, Riyadh, Saudi Arabia

**Keywords:** Bioequivalence trials, Clinical trials, Generic drugs, Study design

## Abstract

**Background:**

This retrospective analysis aimed to comprehensively review the design and regulatory aspects of bioequivalence trials submitted to the Saudi Food and Drug Authority (SFDA) since 2017.

**Methods:**

This was a retrospective, comprehensive analysis study. The Data extracted from the SFDA bioequivalence assessment reports were analyzed for reviewing the overall design and regulatory aspects of the successful bioequivalence trials, exploring the impact of the coefficient of variation of within-subject variability (CVw) on some design aspects, and providing an in-depth assessment of bioequivalence trial submissions that were deemed insufficient in demonstrating bioequivalence.

**Results:**

A total of 590 bioequivalence trials were included of which 521 demonstrated bioequivalence (440 single active pharmaceutical ingredients [APIs] and 81 fixed combinations). Most of the successful trials were for cardiovascular drugs (84 out of 521 [16.1%]), and the 2 × 2 crossover design was used in 455 (87.3%) trials. The sample size tended to increase with the increase in the CVw in trials of single APIs. Biopharmaceutics Classification System Class II and IV drugs accounted for the majority of highly variable drugs (58 out of 82 [70.7%]) in the study. Most of the 51 rejected trials were rejected due to concerns related to the study center (*n* = 21 [41.2%]).

**Conclusion:**

This comprehensive analysis provides valuable insights into the regulatory and design aspects of bioequivalence trials and can inform future research and assist in identifying opportunities for improvement in conducting bioequivalence trials in Saudi Arabia.

## Background

The development of generic drugs has played a pivotal role in increasing patient access to affordable medications (90% of the prescribed drugs in the United States [U.S.] are dispensed as generics with $2.4 trillion dollars cost savings in the past decade), and has been associated with improved patient adherence and clinical outcomes [1–6]. These impacts were anticipated given the abbreviated nature of developing and approving generic drugs (i.e. generic drug manufacturers are precluded from replicating pre-clinical and clinical development programs of the corresponding brand-name drugs) [7–10]. From a clinical perspective, generic drugs are only anticipated to achieve equivalence to their corresponding brand-name drugs in terms of the rate and extent of absorption (i.e. bioequivalence) [7–10]. The in vivo assessment of bioequivalence is performed in the context of bioequivalence trials that are conducted mostly in a small sample of at least 24 healthy volunteers (*n* = 12 per group), and designed in a crossover fashion to demonstrate equivalence in several pharmacokinetic parameters using pre-defined bioequivalence margins [7–10]. However, alternative design aspects/settings might be acceptable by regulators in certain conditions (e.g. drugs with a long plasma elimination half-life, drugs with highly variable pharmacokinetic parameters, or when recruiting healthy volunteers is considered unethical for safety reasons) [7–10]. Notwithstanding the submission of thousands of bioequivalence trials to regulatory agencies, little is known about their designs, the extent of alternative design choices were used, and to what extent bioequivalence was demonstrated as judged by regulators both internationally and in Saudi Arabia.

Most reviews on published bioequivalence trials (or those assessed by regulators) were directed towards re-analyzing their original findings (e.g. pharmacokinetic parameter estimation, bioequivalence testing) [11–13]. A comprehensive design characterization was only provided by one study which covered bioequivalence trials that were assessed by the Ministry of Food and Drug Safety in South Korea [14]. However, the latter study excluded trials that did not demonstrate bioequivalence and those conducted for fixed-combination products. Additionally, the study did not provide data on some important bioequivalence design aspects (e.g. country of the trial center, patient ages, body measurements, number of patients who completed the study, details about the nature of replicated trials). None of the published reviews was conducted using data on bioequivalence trials from the Middle East in general and in Saudi Arabia in particular. In Saudi Arabia, there is a lack of knowledge about bioequivalence data of the approved generic drugs in the Saudi health market [15, 16]. Studies have shown that medical representatives have been the major source of clinical information about generic drugs for physicians, and only small proportion relies on published bioequivalence trials [17, 18]. A panel of Saudi experts also recommended providing more data about bioequivalence trials for the approved generic drugs in the Saudi Arabia [15, 18].

Since the establishment of the new electronic recording system of bioequivalence trials at the Saudi Food and Drug Authority (SFDA) in 2017, hundreds of bioequivalence trials have been reviewed by the SFDA’s evaluators for the approval of generic drugs in Saudi Arabia. The review process has been based on the SFDA Guidelines for Bioequivalence and on the SFDA’s Product Specific Bioequivalence Guidance [7, 8]. This study was aimed to provide comprehensive review of all bioequivalence trials that were assessed by the SFDA since 2017 from a design and regulatory perspective.

## Methods

### Study design and data source

This was a retrospective, comprehensive analysis study. The SFDA generates assessment reports for bioequivalence trials if they were included in generic drug registration applications (one report for all bioequivalence trials per drug application). In each assessment report, all details per trial about the design, technical, and logistic aspects are added, with feedback on each aspect from the SFDA’s scientific evaluators. Each report ends with a decision on whether bioequivalence was demonstrated, was not demonstrated, or whether a decision could not be reached due to concerns related to the design or conduct of the trial. Data for trials since 2017 were extracted by the study team members using several data collection sheets. Approval from the SFDA Institutional Review Board was granted (the data are confidential and not available in the public domain of the SFDA).

### SFDA requirements for bioequivalence testing

Based on the SFDA guidelines [7], the successful demonstration of bioequivalence is conditional mainly on the following:

- *Achieving statistical equivalence in the targeted pharmacokinetic parameters*: The 90% confidence interval (CI) for the ratio of the test generic drug vs. reference brand-name drug must be contained within 80.0 to 125.0% equivalence margins in the targeted pharmacokinetic parameters, mainly the area under the plasma concentration curve from administration to last observed concentration at time t (AUC_0-t_) and the maximum plasma concentration (C_max_). Stricter equivalence margins are required for drugs with a narrow therapeutic index. However, more lenient margins (up to a maximum of 69.84 to 143.19 in C_max_) might be acceptable with highly variable drugs, i.e., CVw > 30%.
- *Choosing the right design aspects*: The typical 2-sequence, 2-period, crossover design (i.e., 2 × 2) under fasting conditions is generally requested for immediate-release oral formulations, unless the brand-name drug is administered under fed conditions. Two 2 × 2 bioequivalence trials (one under fasting and one under fed conditions) are required for specific dosage forms (e.g., modified-release dosage forms, microemulsions, and solid dispersions). Alternative designs (e.g., replicate and parallel trial designs) could be considered in highly variable drugs or in drugs with a long elimination half-life (t_1/2_). Other considerations about alternative design aspects are provided in the SFDA Guidelines for Bioequivalence and by the SFDA Product Specific Guidelines.
- *Compliance with quality and other regulatory standards*: Assessing bioequivalence findings cannot be completed without meeting certain quality and regulatory standards. Examples of these standards are the selection of the production batch, specifications of critical quality attributes, whether the trial was conducted in a center accredited by the SFDA, and the choice of bioanalytical methodology.

### Study outcomes and statistical analysis

Our review of bioequivalence trials that were assessed by the SFDA was conducted for three outcomes: reviewing the overall design and regulatory aspects of bioequivalence trials that were deemed successful by the SFDA in demonstrating bioequivalence, exploring the nature of within-subject variability in these trials and its relevance to other design aspects (e.g. the trial sample size), and providing an in-depth assessment of bioequivalence trial submissions that were deemed insufficient in demonstrating bioequivalence.

The overall review of the successful bioequivalence trials covered the following: the biopharmaceutics classification system (BCS), the therapeutic class of the active pharmaceutical ingredient (API) which was determined according to the World Health Organization Anatomical Therapeutic Chemical codes (ATC), the dosage form, the safety index (wide vs. narrow therapeutic index), the trial center, the trial design (2 × 2 crossover, replicate or parallel designs), the study condition (fasting vs. fed), masking study participants (blinding vs. open-label designs), the washout period, the sample size, the choice of equivalence margins, the coefficient of variation of within-subject variability (CVw), age, sex, and body mass index. The review was conducted at the single-API and fixed-combination levels.

The extracted CVws from the accepted trials (for both single APIs and fixed combinations) were assessed in relation to the trial sample size , trial design, and the different BCS classes (the recent Korean review found that highly variable drugs are mostly BCS Class II or IV [i.e. drugs with poor solubility]) [14]. Reasons for rejecting the submission of bioequivalence trials or for rejecting their results were explored in addition to the success rate of study centers per country. All study outcomes were analyzed descriptively using RStudio Version 2022.12.0.

## Results

**Fig. 1:**
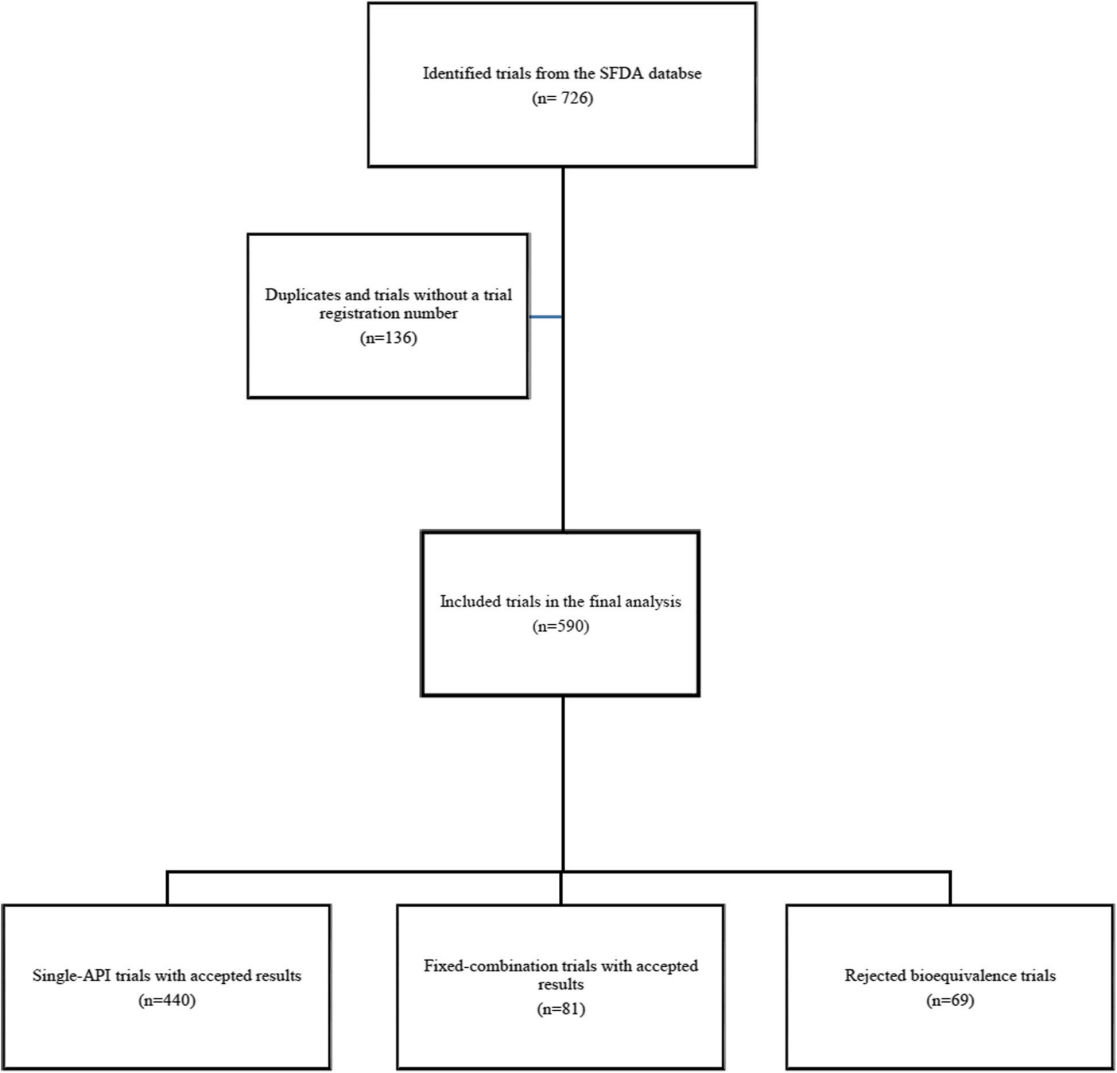
The process of selecting the included bioequivalence trials

A total of 590 bioequivalence trials were included in the study (Fig. 1). Bioequivalence was demonstrated in 521 trials (440 and 81 trials for single-API and fixed combination drugs; respectively), which were used to evaluate bioequivalence for 184 unique APIs and 36 unique fixed combinations. The remaining 69 of 590 were rejected either due to failed submissions of marketing authorization applications or due to the failure of a trial to demonstrate bioequivalence. Of the included trials, 240 (40.7%) were conducted ≥ 2017 (the trial date was not available for one trial). Almost two-third of 590 trial were conducted using Jordanian and Indian populations (237 and 203 trial centers; respectively), and 98 trials (16.6%) were conducted in European centers (only one study was conducted in Saudi Arabia).

### The design of the successful bioequivalence trials

**Fig. 2:**
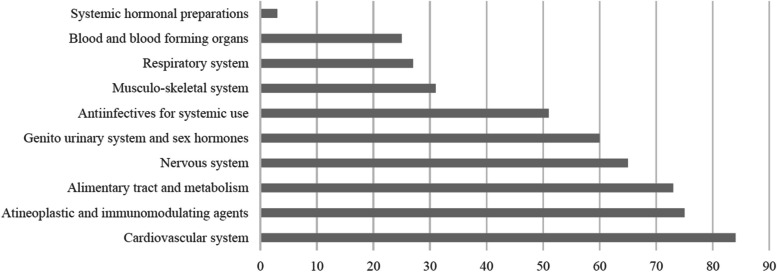
The most common therapeutic classes of successful trials

Most of the 521 successful trials were conducted for single APIs/fixed-combinations of the cardiovascular system (84 of 521 [16.1%]), antineoplastic and immunomodulating drugs (75 [14.4%]), drugs for alimentary tract and metabolism (73 [14.0%]), and drugs for the nervous system (65 [12.5%]), Fig. 2. More than half of single-API trials were BCS Class I drugs, and most BCS combinations in the fixed-combinations were BCS Class I and II combination and Class I and III combination (15 combinations each). Almost two-third and all of single-API and fixed-combination trials were conducted for tablet and capsule dosage forms; respectively (Table 1). A large proportion (79.0%) was conducted under fasting conditions (85.5% of these trials were conducted for immediate-release dosage oral dosage forms), and all trials. except for six, were conducted for drugs with a wide therapeutic index.

**Table 1:** Design aspects of the successful trials

**Design aspect**	**Single-API preparations** Number of trials=440	**Fixed-Combination preparations = 72 dual-fixed and 9 triple-fixed combinations (*n*=167 APIs)**
**BCS class (n [%])**	**436**	**167**
**BCS Class I**	104 (23.9)	42 (25.2)
**BCS Class II**	220 (51.1)	58 (34.7)
**BCS Class III**	59 (13.5)	43 (25.7)
**BCS Class IV **	50 (11.5)	24 (14.4)
**Dosage forms (n [%]) **		
***Oral dosage forms***	***435***	**80**
**Immediate-release forms**	369 (84.8)	72 (90.0)
**Modified-release forms **	66 (15.2)	8 (10.0)
***All dosage forms***	**438 **	
**Tablets **	333 (76.0)	72 (88.9)
**Capsules **	95 (21.7)	4 (5.0)
**Liquid oral dosage forms**	7 (1.6)	1 (1.2)
**Topical preparations **	2 (0.5)	0 (0.0)
**Inhalers**	1 (0.2)	1 (1.20)
**Drug toxicity index (n [%])**	**438**	**81**
**Wide therapeutic index **	432 (98.6)	81 (100.0)
**Narrow therapeutic index**	6 (1.4)	0 (0.0)
**Trial design**		
**2 x 2 crossover design **	383 (87.0)	72 (88.9)
**Replicate design **	49 (11.0)	9 (11.1)
**Parallel design **	9 (2.0)	
**Blinded design **		
**Yes **	14 (3.2)	1 (1.2)
**No **	426 (96.8)	80 (98.8)
**Washout period (days) (median [IQR** ^**a**^ **])**	**0 to 60 (7 [7]) **	**3 to 35 (14 [14])**
**Trial condition**		
**Fasting (n [%])**	351 (79.8)	61 (75.0)
**Fed (n [%]) **	89 (20.2)	20 (25.0)
**Coefficient of variation (median [IQR]) **	**5.1 to 123.3 (20.0 [12.2])** **432**	**6.0 to 47.9 (20.89 [12.8])** **80**
**Trials with CVw ≤30% (n [%])**	350 (81.0)	61 (76.3)
**Trials with CVw>30% (n [%])**	82 (19.0)	19 (23.7)
**Using wider equivalence margins **		
**2 x 2 crossover design **	2/383	0/72
**Replicate design**	24/49	1/9
**Health measurements of trial participants**		
**Minimum age (median [range])**	19 (18 to 52)	19 (18 to 24)
**Maximum age (median [range])**	44 (18 to 87)	45 (34 to 76)
**Body mass index (median [range]) **	23.8 (19.6 to 27.8)	23.5 (18.5 to 25.5)

^a^ *IQR* Interquartile range

The typical 2 × 2 crossover design was used in 455 (87.3%) trials. The replicate design was used in 58 trials (wider C_max_ equivalence margins were used in 25 of 58 replicate trials). Nine trials were designed as randomized, parallel design trials (four trials for generic forms of fingolimod hydrochloride, and five for enzalutamide, toremifene, entecavir, and terifunormide). The range of t_1/2_ for drugs in these nine parallel trials was 4 to 20 days. The range of the sample size was 12 to 216. The median sample size for the single APIs was 36, and per trial design, the parallel-design trials had the highest median number of study participants (*n* = 71), followed by the replicate and 2 × 2 crossover trials (40 and 35; respectively). Similarly, the fixed-combination trials had a median of the sample size (*n* = 36), and almost comparable medians per trial design (*n* = 39 and 36 for the replicate and 2 × 2 crossover trials).

### CVw, sample size, trial design and BCS classification

**Fig. 3:**
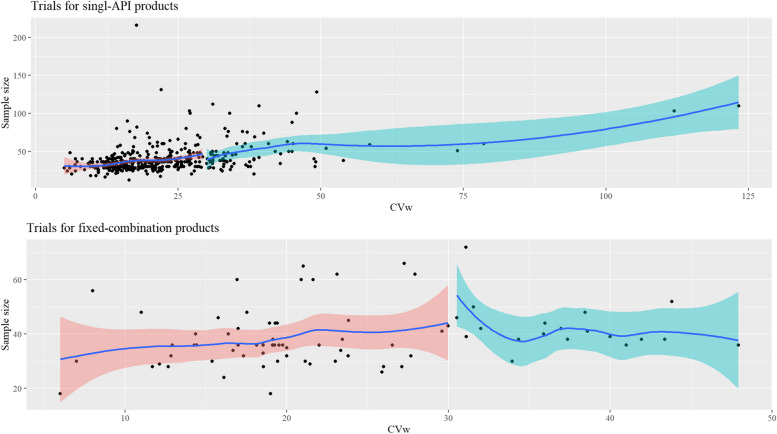
CVw vs. trial’s sample size.  CVw≤30%: Trials of active pharmaceutical ingredients with CVw≤30%.  . CVw>30%: Trials of active pharmaceutical ingredients with CVw>30%

Figure 3 shows that the sample size tended to increase with the increase in CVw in bioequivalence trials of single APIs. The median sample size in trials of highly variable, single APIs was higher vs. the median in trials of drugs with CVw of ≤30%. This trend was not observed in trials of the fixed-combinations (Fig. 3); however, the median sample size in trials of fixed-combinations with a highly variable API was slightly higher vs. those low-CVw APIs (40 vs. 36; respectively).

**Fig. 4:**
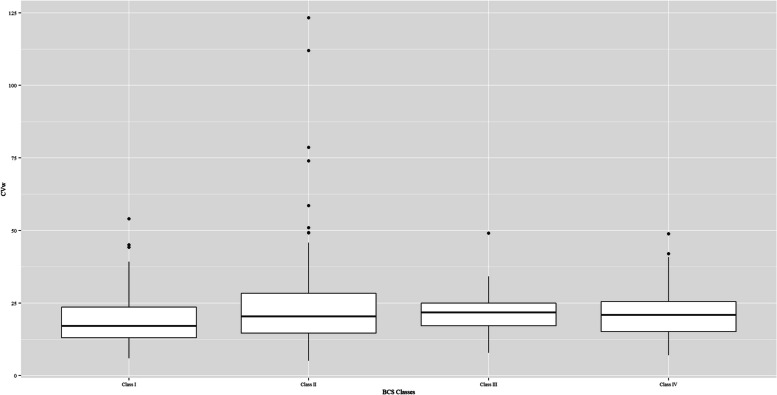
The distribution of CVws per BCS class. Class I: Trials with BCS Class I active pharmaceutical ingredients. Class II: Trials with BCS Class II active pharmaceutical ingredients. Class III: Trials with BCS Class III active pharmaceutical ingredients. Class IV: Trials with Class IV active pharmaceutical ingredients

Trials with replicate design had the highest median CVw for both the single APIs (33.5% vs. 18.9% vs. 16.3% compared with the 2 × 2 crossover and parallel trials; respectively) and the fixed combinations (31.1% vs. 19.8% compared with the 2 × 2 crossover trials). BCS Class II and IV drugs accounted for most of the highly variable drugs (58 of 82 [70.7%] of the highly variable drugs in the successful trials were BCS Class II and IV drugs). Figure 4 shows the distribution of CVw at the BCS class level in the single-API trials.

### The rejected bioequivalence trials

Justifications for the SFDA rejection was available for 51 of 69 (73.9%) rejected trials (Table 2). Most of these 51 trials (*n* = 21 [41.2%]) were rejected due to concerns related to the study centers (e.g. the center was not accredited by the SFDA; the center was suspended by the SFDA or a reference regulatory authority [e.g. US FDA or EMA]). Ten trials (19.6%) failed do demonstrate bioequivalence, and 17 had major regulatory or design concerns or concerns related to the quality of the APIs (Table 2).

**Table 2:** Justifications for rejecting 51 trials

**Reasons for rejecting BE studies**	**51**
**Concerns related to the study center (e.g. the center was not a SFDA accredited center, the center was suspended by the SFDA, EMA or FDA) **	**23**
**Bioequivalence was not concluded**	**10**
**Design and conduct concerns:**	**6**
- Sub-optimal selection of the reference product	2
- In appropriate selection of the lower limit of quantification	1
- Sampling schedule/drug quantification did not cover a plasma concentration time curve long enough to calculate reliable estimates	1
- The anticipated study condition (fast/fed) was not followed	2
- Suboptimal analytical procedures	1
**Quality concerns:**	**3**
- Concerns about the quality of active pharmaceutical ingredients	3
**Regulatory concerns:**	**8**
- Rejection of a biowaiver submission	4
- Non-compliance with the SFDA submission standards	3
- The bioequivalence study was conducted for a strength that was not intended for SFDA registration	1

There were four unsuccessful attempts to obtain waivers for demonstrating bioequivalence through in-vivo testing, commonly known as biowaivers. The first was denied because the request to register a 20 mg generic form of rosuvastatin relied on previous in-vivo bioequivalence data from the unregistered higher strength of 40 mg (i.e., additional strength waiver requests for generic drugs might be acceptable if the maximum strength is approved by the SFDA). Additionally, two other requests for biowaivers were made for generic forms of two drugs, ibuprofen and mirtazapine, which are not classified as BCS Class I (the SFDA only accepts biowaiver requests for BCS Class I drugs). The fourth and final rejected request was for a generic form of dabigatran etexilate mesylate, which failed in the in-vitro comparative dissolution testing—a requirement by the SFDA for biowaiver applications.

**Fig. 5:**
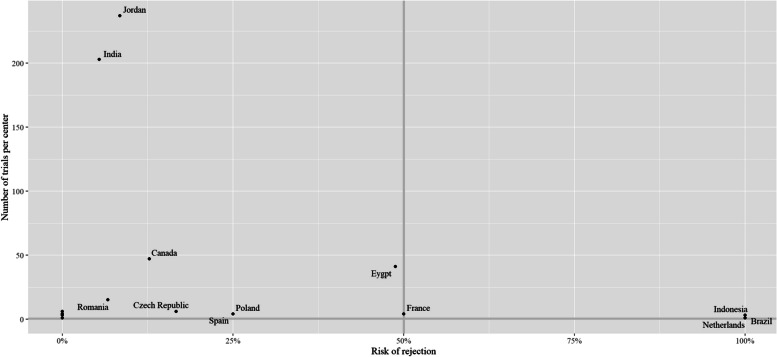
The risk of rejecting bioequivalence trials per country

 Figure 5 illustrates the risk of rejecting a bioequivalence trial per country. There were five countries with a number of conducted bioequivalence trials of higher than 10: Jordan (*n* = 237), India (203), Canada (*n* = 47), Egypt (*n* = 41), and Romania (*n* = 15). The highest risk of rejection among these five countries was in Egypt (48.8%), followed by Canada (12.8%), and Jordan (8.4%). The SFDA rejection decision in the Egyptian trials was driven by one of the three trial centers, which led to its suspension by the SFDA.

## Discussion

For the support of the marketing authorization of generic drugs in Saudi Arabia, we found that the majority of submitted bioequivalence trials were accepted by the SFDA. Most were designed in the conventional 2 × 2 crossover format, with a focus on immediate-release dosage forms. These trials were primarily conducted in Jordan and India, with most drugs exhibiting low CVw. Additionally, a significant proportion of the trials involved BCS Class II drugs, and the therapeutic categories most frequently tested were drugs for the cardiovascular system, and antineoplastic agents and immunomodulatory drugs. Studies involving single APIs with high CVw tended to require larger sample sizes, with BCS Class II and IV drugs accounting for the majority of high CVw drugs. Finally, our findings indicate that conducting trials at centers not accredited by the SFDA or suspended by other stringent authorities is likely to result in trial rejection.

Several design aspects of the accepted bioequivalence trials in our study share similarities with those submitted to other regulators [13, 14, 19]. This similarity can be attributed to the common adoption of a 2 × 2 crossover design, immediate-release oral dosage forms, and drugs for the cardiovascular system as the most commonly targeted therapeutic class. Our study identified six parallel trials, which typically had a larger sample size than other designs (median sample sizes were 70, 40, and 35 for parallel, replicate, and crossover trials; respectively). The range of sample sizes in our study was wider compared to those reported in published reviews (12 to 216 versus 12 to 170) [13, 14]. Our study also revealed that an increase in sample size was necessary to accommodate an increase in CVw for trials with single APIs. Notably, we observed that the replicate design had the highest median CVw, as expected. Consistent with previous research, which found that CVw was influenced by solubility-limited absorption and/or low permeability, our results indicated that BCS Class II and IV drugs accounted for the highest variability [14, 20].

The age range of trial participants in our study was wider (18 to 87 years) than those reported in previous reviews (18 to 60 years) [13, 19]. The SFDA might accept an age limit higher than 60 years if adequately justified by the marketing authorization holder. Almost two-third of the trials in our study were conducted under fasting conditions, similar to trials conducted in South Korea [19]. Only six trials were conducted for narrow-therapeutic index drugs, and no data from published reviews were available for these drugs. Only one study was conducted in Saudi Arabia, with the majority of trials conducted in Jordanian and Indian populations. Further studies are needed to explore opportunities and to identify gaps in conducting bioequivalence in Saudi Arabia, and to determine the extent to which bioequivalence results can be extrapolated directly to the Saudi population as a previous study found differences in fed-between-population pharmacokinetic parameters [21].

The study findings indicated a potential lack of understanding or insufficient knowledge regarding SFDA requirements and guidelines for the design and conduct of bioequivalence trials. For instance, conducting trials at centers not accredited by the SFDA resulted in a rejection of 23 bioequivalence trials, and two trials were rejected because the approved brand-name drug was not chosen as the reference drug. There might also be insufficient awareness for the conditions of accepting applications for biowaivers. The SFDA published a comprehensive product-specific bioequivalence guideline in 2022 and they have advised marketing authorization holders to seek for an SFDA scientific advice during the design, conduct or submission stages if further clarification is needed [8]. The number of SFDA bioequivalence scientific advice letters/meetings increased in the first two months of 2024 in comparison with those of 2023 (43 vs. 15; respectively). The SFDA is in the process of updating its guideline for biowaivers to include more details with illustrative examples. These initiatives are expected to facilitate the development of generic drugs and reduce the number of failed or suboptimal applications for demonstrating or waiving bioequivalence [22].

Our study provides a comprehensive assessment of bioequivalence trials submitted to a regulator for marketing authorizations of generic drugs, making it among the few studies of its kind and the first in the Middle East. We presented a summary of the design, conduct, and regulatory aspects of these trials, investigated the association of CVw with certain design factors, and analyzed trials rejected by the SFDA in an effort to support the implementation of these trials in the region. However, our study had several limitations. Trials submitted for marketing authorization prior to the introduction of the new electronic recording system for bioequivalence trials in 2017 were not included in our analysis. Additionally, we did not evaluate the selection of reference drugs in these trials or explore the submission of marketing authorization applications for biowaivers. Future studies that address these aspects would complement the findings of our study.

## Conclusions

In conclusion, this study provides valuable insights into the design, conduct, and regulatory aspects of bioequivalence trials submitted to the SFDA for marketing authorization of generic drugs in Saudi Arabia. The findings of this study highlight the need for further research to explore opportunities and identify gaps in conducting bioequivalence trials in Saudi Arabia.

## Data Availability

The datasets generated during and/or analysed during the current study are not publicly available due to confidentiality but are available from the corresponding author on reasonable request.
